# Accelerating harm reduction interventions to confront the HIV epidemic in the Western Pacific and Asia: the role of WHO (WPRO)

**DOI:** 10.1186/1477-7517-5-26

**Published:** 2008-08-05

**Authors:** Fabio Mesquita, David Jacka, Dominique Ricard, Graham Shaw, Han Tieru, Hu Yifei, Katharine Poundstone, Madeline Salva, Masami Fujita, Nirmal Singh

**Affiliations:** 1 World Health Organization, Regional Office for Western Pacific (WPRO), HIV/AIDS and STI Unit, Manila, Philippines; 2 World Health Organization, Hanoi, Viet Nam; 3 World Health Organization, Vientiane, Lao PDR; 4 World Health Organization, Phnom Penh, Cambodia; 5 World Health Organization, Kuala Lumpur, Malaysia; 6 World Health Organization, Beijing, PR China; 7 World Health Organization, Manila, Philippines

## Abstract

The epidemic of HIV/AIDS linked to injecting drug usage is one of the most explosive in recent years. After a historical epicentre in Europe, South and North America, at present it is clearly the main cause of dissemination of the epidemic in Eastern Europe and some key Asian countries. Recently, 10 African countries reported the spread of HIV through people who inject drugs (PWID), breaking one of the final geographical barriers to the globalization of the epidemic of HIV among and from PWID.

Several countries of the Asia and Pacific Region have HIV epidemics that are driven by injecting drug usage. Harm reduction interventions have been implemented in many countries and potential barriers to implementation are being overcome. Harm reduction is no longer a marginal approach in the Region; instead, it is the core tool for responding to the HIV/AIDS epidemic among PWID. The development of a comprehensive response in the Region has been remarkable, including scaling up of needle and syringe programmes (NSPs), methadone maintenance treatment (MMT), and care, support and treatment for PWID. This development is being followed up by strong ongoing changes in policies and legislations. The main issue now is to enhance interventions to a level that can impact the epidemic.

The World Health Organization (WHO) is one of the leading UN agencies promoting harm reduction. Since the establishment of the Global Programme on AIDS, WHO has been working towards an effective response to the HIV epidemic among PWID. WHO's work is organized into a number of components: establishing an evidence base; advocacy; development of normative standards, tools and guidelines; providing technical support to countries; ensuring access to essential medicines, diagnostics and commodities; and mobilizing resources.

In this paper, we trace the course of development of the HIV/AIDS epidemic among and from PWID in the Western Pacific and Asia Region (WPRO) as well as WHO's role in supporting the response in some of the key countries: Cambodia, China, Lao PDR, Malaysia, the Philippines and Viet Nam.

## Background

Worldwide, around 13 million people inject drugs [1]. The first cases of HIV related to injecting drug use were reported in the United States of America in 1982 in the *MMWR *bulletin from the Centers for Disease Control and Prevention (CDC), Atlanta [2]. As of September 1982, 13% of the AIDS cases in United States of America were already people who inject drugs [2].

By the late 1980s and 1990s, concentrated epidemics of HIV in PWID were restricted to North and South America and Europe. Since the beginning of this century, explosive epidemics of HIV have occurred among PWID in Eastern Europe and in many countries of South, Central and South-East Asia. Cases of HIV infection within the community of PWID were recently reported from 10 African countries, disrupting the geographical barrier for a globalized phenomenon [3].

Harm reduction is a new name for an old concept. One of the first harm reduction activities was described in Great Britain in 1926, when Sir Humphrey Rolleston, President of the Royal College of Physicians, proposed the use of morphine or heroin for the treatment of opium addiction. This decision was the beginning of a pragmatic and humanitarian approach to the treatment of drug addiction [4].

In 1984, to control an outbreak of hepatitis B in the Netherlands and pressured by the Association of Drug Addicts (Junkies' Union), the health authorities of the City of Amsterdam implemented one of the first known needle exchange programme with a very successful outcome [5]. When the first cases of HIV transmitted by the sharing of needles were recognized in 1985, the technology developed in Amsterdam was applied, again with good results[5]. This approach has subsequently been proven to be extremely effective in controlling HIV epidemics among PWID [6-9].

The offer of treatment for drug dependence has proven to be a very effective tool in controlling the HIV/AIDS epidemic arising from injecting drug use. From abstinence-based treatment, to detoxification or self-support treatment (such as Narcotics Anonymous), all these measures have some impact on the epidemic [10]. However, substitution therapy, in the case of opioids, is probably the most effective and scientifically proven strategy to treat drug dependence [10,11].

There is no published information on when methadone was first used to treat heroin dependence. Apart from reducing or eliminating illicit heroin and other drug use as well as other benefits, methadone maintenance treatment (MMT) reduces the level of involvement with crime associated with opioid use [10,11]. Besides methadone, other opioid substitution therapies (OSTs) are available such as buprenorphine (both included in the WHO Model List of Essential Drugs in 2005) [12].

Another important strategy to control the HIV/AIDS epidemic among PWID is the offer of antiretroviral therapy (ART) for those in need [13-15]. A comprehensive package of harm reduction interventions (including needle and syringe programmes [NSPs], substitution therapy [ST], and care, support and treatment for PWID) is crucial for controlling the HIV/AIDS epidemic and is being implemented in many countries. It is now supported by all UN agencies [16].

Since the establishment of the Global Programme on AIDS, WHO has been working in a strategic and systematic way in the field of HIV/AIDS and drug use. WHO is one of the leading UN agencies promoting harm reduction and the main interlocutor with the health system involvement in harm reduction. Its work is organized into a number of components: establishing an evidence base; advocating for effective policies and programmes; developing normative standards, tools and guidelines; supporting countries in implementing programmes; ensuring access to essential medicines (such as methadone and buprenorphine), diagnostics and commodities; and mobilizing resources [17]. The Organization has officers working on harm reduction in almost all the WHO Regions who focus on controlling epidemics caused by injecting drug use. In principle resolutions are based on available evidence and backed by science and experts opinions, but as a multilateral organization there are also political elements that can influence on the decision making process.

### The Western Pacific and Asia Regions

Although national HIV prevalence levels are still very low in the Asia-Pacific region, the huge population sizes of many Asian countries mean that very large absolute numbers of people are being infected each year with HIV [18]. Urgent responses are required; effective responses in some areas of the epidemic by countries such as Thailand and Cambodia have shown how much can be done [19,20]. Unlike in Africa, the Asian epidemics are concentrated in identifiable high-risk groups (primarily those involving PWID who share needles, sex workers and men who have sex with men). Hence, HIV in the Asia region could be controlled if these high-risk groups are targeted with specific interventions [21,22].

Many countries in the Asia-Pacific region face difficult public policy and legislative problems with regard to sex work, homosexuality and drug use. In addition, widespread poverty, and a general lack of access to effective health-care services by the poor and disadvantaged in both rural and urban areas means that the challenges of developing targeted intervention programmes, and ensuring coverage of vulnerable groups, are particularly acute [20].

The WHO Western Pacific Region comprises 37 countries from the Mekong River Valley in Asia to countries in the Pacific. Many of the epidemics in the Region are currently driven by needle-sharing among PWID. The epidemic of HIV is clearly driven by injecting drug use in three of the countries (China, Malaysia and Viet Nam). China accounts for 75% of the total population of the Region. Three other countries in the Region are beginning to build a comprehensive response to limit the burden of the HIV epidemic related to injecting drug use (Cambodia, Lao PDR and the Philippines). The other 31 countries are considered very Low Prevalence countries (with the exception of Papua New Guinea considered as the only country in the region with a generalized epidemic, but with a clear sexually transmitted epidemic). In the present paper we concentrate the information in the 6 key countries for the subject.

### Cambodia

#### The epidemic in Cambodia

The first case of HIV infection was notified in Cambodia in 1991. By 2003, approximately 123,000 people were living with HIV including 57,000 women and 9,000 children out of a total population of 14,071,000 [23,24]. An estimated 19,800 adults had developed AIDS by 2005 [25].

National prevalence of HIV among those aged 15–49 years has declined from 1.2% in 2003 to 0.9% in 2006 [26] with HIV transmission occurring primarily through sexual contact [23]. In June 2007, HIV sentinel surveillance (HSS)-based data from the Center for HIV/AIDS/Dermatology/STIs (NCHADS) revealed that in 2006 the number of people living with HIV/AIDS (PLHA) was approximately 67,200, showing that the number of PLHA has decreased over time [26].

In 2004, about 20,000 people were using amphetamine-type stimulants (ATS) and 2,500 were heroin users, of whom 1,750 might have been PWID [27]. Local nongovernmental organization (NGO) reports using small sample sizes put the HIV prevalence among PWID in the capital, Phnom Penh, at around 15%, and of ATS users at around 5% or less [28]. Knowledge of HIV transmission through shared needles/syringes is poor among PWID [29]. Consequently, patterns of the epidemic seem to be changing from one driven by sexual contact to one mixed with injecting drug use.

#### Response to the epidemic in Cambodia

Cambodia developed a policy for mounting a strategic response to harm reduction in 2003. The HIV/AIDS National Strategic Plan of the National AIDS Authority (NAA) explicitly promotes harm reduction as an evidence-based intervention [30]. The drug control master plan for Cambodia, developed by the National Authority for Combating Drugs (NACD), refers to the need to implement a comprehensive approach to HIV/AIDS [31].

A memorandum of understanding (MoU) was signed between the NACD and NAA in 2004 to collaborate in preventing drug-related HIV/AIDS. An illicit drug-related HIV and AIDS working group (DHAWG) was then established to integrate HIV/AIDS into the full range of illicit drug-related activities nationwide and now meets quarterly.

Capacity building for harm reduction has been undertaken of several provincial NGOs and Government social service agencies. Two local NGOs in Phnom Penh have been operating comprehensive harm reduction community outreach and drop-in centre activities – including NSPs – under NACD authorization since 2005. Cambodia now has at least 11 centres for the treatment and rehabilitation of drug users located around the country [32]. However, service capacity in all such centres is extremely limited according to Multi Agencies Rapid Assessment of treatment and rehabilitation centres in Cambodia. Phnom Penh conducted in December of 2007 [unpublished]. Minimum standards for such centres have now been drafted for Government approval.

In 2008, the Government announced its intention to establish a methadone maintenance programme in Phnom Penh (document N° DG-DHS). Planned for September 2008, referrals will be established to services for voluntary counselling and testing (VCT), sexually transmitted infections (STIs) and tuberculosis (TB), prophylaxis and treatment of opportunistic infections (OIs)/ART as well as emergency medical assistance. NCHADS is also developing detailed plans to establish several VCT demonstration sites in the capital to attract drug users; this approach will then be scaled up nationwide. In 2008, NCHADS also plans to commence referral for prisoners to VCT, and drug treatment and rehabilitation centres nationwide.

#### The role of WHO in Cambodia

Since its support to a qualitative assessment of drug use in 2004 in collaboration with the US CDC, WHO in Cambodia has become the co-lead agency with the United Nations Office on Drugs and Crime (UNODC) for drug use in the Joint UN Theme Group on HIV/AIDS [33]. WHO's work includes technical assistance to develop and implement a continuum of care for drug users in all settings in Cambodia using evidence-based international good practices.

In collaboration with a range of partners, WHO (i) provides support for the operationalization of the DHAWG and associated costed National Strategic Plan for Drug Use and HIV/AIDS; (ii) provides technical assistance for the NSP policy, guidelines, standard operating procedures (SOPs) and implementation plan (with related training in 2006); (iii) provides technical support to establish an MMT programme; (iv) gives technical advice for the development of policies, guidelines and SOPs related to drug dependency detoxification and treatment, and related resource mobilization for both community and closed settings; (v) supports linkages between the NACD, NCHADS, National Programme for Mental Health (NPMH) and NGOs working with drug-using communities for an effective response to the epidemic; (vi) supports communication initiatives including the development of guidelines in the Khmer language targeting health, social and education service providers, law enforcement personnel and parents, as well as four harm reduction video educational interventions for PWID, entertainment workers, ATS users and inhalant/solvent users; (vii) provides technical assistance to the Government and its NGO partners for resource mobilization, such as the Global Fund and the Swedish International Development Agency (SIDA).

### China

#### The epidemic

China sits between the "Golden Triangle" and "Golden Crescent" – two of the three biggest opiate suppliers in the world – and has seen a significant surge in the number of illicit drug users since the 1980s. Heroin is the main illicit drug used in China, with amphetamine and polydrug use on the rise.

China's first case of HIV was reported in 1985 and first case due to injecting drug use in 1989 [34]. By December 2007, there were an estimated 700,000 PLHA in China [35]. The national HIV prevalence is 0.05% (range 0.04% to 0.07%) [36]. Prevalence among PWID increased from 1.95% in 1996 to 6.48% in 2004, and is rising and spreading from these groups to the general population [36].

PWID continue to engage in high-risk behaviours. Recently published national surveillance data show that 40% of participating PWID reported needle-sharing [35]. Estimated data indicate a focused spread of HIV infection among PWID. At the end of 2005, there were about 288,000 drug users living with HIV/AIDS, accounting for 44.3% of the total estimated HIV cases [37]. The epidemic is concentrated in Yunnan, Xinjiang, Guangxi, Guangdong, Guizhou, Sichuan and Hunan provinces, with each province having an HIV prevalence of more than 5% among PWID and over 10,000 HIV-positive PWID. Together, they account for 89.5% of all people infected through injecting drug use[36]. As of 30 December 2005, 1.16 million drug users were registered with the Ministry of Public Security (MPS) [37].

#### Response to the epidemic in China

The unique pattern of the HIV/AIDS epidemic in China – high HIV infection rate among PWID and former plasma donors (FPDs), but a relatively low overall infection rate in the country – provides a window period for taking action [38]. China has achieved remarkable national progress in its comprehensive response to HIV/AIDS. The first and second five-year Action Plans (2001–2005 and 2006–2010) were formulated; the second aimed to cover no less than 90% of most-at-risk populations and vulnerable migrants with effective prevention interventions. By 2010, drug maintenance treatment clinics should be set up to provide services for no less than 70% of opium users (mainly heroin users) in counties and cities with more than 500 registered drug users. No less than 50% of PWID in the areas implementing NSPs should be provided with clean needles and syringes[39]. In 2006, the State Council issued the AIDS Prevention and Treatment Regulations that include promotion of programmes for drug users such as drug-maintenance treatment and other effective interventions [40]. Although the regulation does not explicitly mention needle exchange programmes, implementation of the approach in 775 sites in 17 provinces indicates the endorsement of this approach in the country [39].

In February 2003, the Ministry of Health (MOH), MPS and the State Food and Drug Administration (SFDA) issued an interim "Opium abusers community-based drug maintenance treatment protocol" to start piloting MMT [41]. In July 2006, the three agencies revised the protocol to support expansion of the MMT programme in 22 provinces [42].

A 2007 evaluation survey conducted in the first phase of eight MMT clinics found a positive change in the self-reported rate of injecting drug use, drug-related illegal offences, employment opportunities and family relations [35]. The average frequency of drug injection declined from 90 to two times per month and self-reported criminal behaviours reduced from 20.7% to about 3.8% [43]. Entry requirements deterred many people from accessing the services, especially migrants and others without the required documents [39]. In 2006, the Government made adjustments to methadone delivery, such as permitting access to methadone clinics anywhere, linkages to other services, mobile methadone provision and waiving of residency documents [39].

By December 2007, China had 503 functional MMT clinics. Cumulatively, over 97,554 drug users had entered the programme. In addition, 45,121 PWID had regularly attended NSPs [44]. These clinics regularly provide free HIV testing and counselling services to all who join the MMT programme. China has set a target of operating around 700 MMT clinics by the end of 2008 [44].

The Chinese government has taken bold steps to scale up HIV testing and counselling, offer free ART to AIDS patients, and expand primary prevention measures such as MMT and NSPs for drug users, and condom promotion for sex workers and men who have sex with men (MSM). The remarkable achievements in such a short period of time indicate that China is strongly committed to limiting the epidemic and maintaining a low HIV prevalence in the future [38].

Although the current policy environment is favourable for harm reduction activities, China faces several difficult challenges in establishing a comprehensive HIV/AIDS response among PWID. These include (i) difficulty in coordination among different bodies; (ii) inadequate implementation of prevention strategies; (iii) increasing the demand for care, support and treatment for PWID to reach all those in need; and (iv) need for effective control of HIV/AIDS transmission inside closed settings.

In China, eligible HIV-positive PWID in the community have access to free ART since 2003 through referral linkages between MMT clinics, hospitals and CDC in China. Over 31 000 adult and paediatric patients have been treated [45]. China is also piloting ART in prisons in Guangxi, Yunan, Sichuan and Hunan. Prisoners on ART are mainly drug users.

#### The role of WHO in China

WHO assists China to confront the challenges it faces, by providing timely information about new approaches and technologies that are later adapted and adopted according to China's needs. WHO works with several key partners on various issues through the UN Theme Group on HIV/AIDS and Drug Use.

Specifically, WHO supports China in the following areas: (i) policy development and advocacy (e.g. supporting pilot NSP and MMT projects); (ii) providing HIV prevention education; (iii) training staff in drug detoxification and drug dependence treatment in closed settings; (iv) providing technical support for the provision of drug dependence treatment from pilot projects to scaling up; (v) advocacy for testing and counselling; (vi) care, support and treatment for PWID; and (vii) monitoring and evaluation of harm reduction projects.

WHO is also working with the Government to change the common perception that drug users are criminals instead of people who are ill and in need of care, support and treatment.

### Lao PDR

#### The epidemic

Lao PDR is experiencing an impressive development in the recent years. According to UNAIDS Report of 2008, Lao PDR is currently losing the status of a considered land-locked country and being fast transformed in a land linked country [46]. A high way being developed by the Asian Development Bank and the Great Mekong Sub-Region Governments (the Mekong Highway Project) is faster optimizing the mobility of the population and the development of the entertainment industry [47].

Lao PDR is a low HIV-prevalence country. To date, the HIV epidemic has been driven by multipartner client-sex worker behaviours, with some data showing that HIV infection may spread as well among MSM [46]. It is estimated that 3700 people are living with HIV in the country, which has a population of 5,924,000. The estimated prevalence is around 0.1% among the 15–49 years' age group [48].

Lao PDR has Myanmar, China (Yunnan), Viet Nam, Thailand and Cambodia as its neighbours and injecting drug use has been reported in all these countries. Lao PDR is in the border of the Golden Triangle which is one of the important sources of opium globally speaking. Lao PDR is also a neighbor of countries that are producing methamphetamines and is therefore being considered as a transit country for methamphetamine and its precursors [46]. The traditional use of opium is still the main drug used in Lao PDR but there are increased reports in the use of methamphetamines and injection of opium and this trend seems to be a recent phenomenon. The national behavioural surveillance survey (BSS) done in 2001 did not indicate any injecting drug use among the sample [49]. The second round of BSS in 2004 indicated that up to 11% of sex workers in one province indicated that they had ever injected drugs[50]. Anecdotal evidence shows that injecting drug use is becoming more common, though the HIV epidemic has not yet clearly started among PWID.

#### Response to the epidemic in Lao PDR

The response to the HIV/AIDS epidemic in Lao PDR started early and has a high level of public commitment. Lao PDR developed a National Strategy and Action Plan 2006–2010, with clear priority targets. A costed action plan forms the basis for the resource mobilization of the country. According to UNAIDS 99.52% of the funds comes from external resources [46]. Global Fund round 6 is one of the important sources of funds to support the response to the epidemic in Lao, which also has funds from USAID, AusAID, SIDA, among others. It is important to notice that Lao PDR included its action plan for HIV/AIDS in their 6^th ^National Socio-Economic Development Plan, elevating the fight against the epidemic for a higher level of development concerns of the country [46].

Recently, with the support of the UN system, the Lao PDR Government set up a Task Force on HIV and Drug Use with the objectives of developing a policy, proposing concrete activities to address the vulnerability of drug users to HIV, and developing guidelines for harm reduction and treatment of drug abuse [51]. This Task Force is jointly chaired by the Lao PDR National Commission for Drug Control and Supervision, and the Ministry of Health, and is composed of different stakeholders enlarging the participation of many different sectors in the response of the HIV/AIDS Epidemics.

Harm reduction for PWID is embedded in the National HIV/AIDS/STI Strategy 2006–2010 [52]. This includes providing a supportive environment for PWID (advocacy, legal and policy framework, review and update of the national policy) and providing PWID with the means to protect themselves from the consequences of drug use, as well as scaling up towards universal access. So far the harm reduction activities are very incipient in the country and rely on outreach work.

An access to ART project was started in 2003, which at the moment covers around 700 patients in two centres of the country. Even if a larger number of people are in need of ART according to estimates, there are no known people in need of ART who are not receiving it. According to the Universal Access Report launched by WHO, UNAIDS and UNICEF in June of 2008, Lao PDR is one of the few countries in the world where the coverage for people in need of ARV bounds 100% [53].

#### The role of WHO in Lao PDR

WHO supports the Task Force technically (with UNODC and UNAIDS) and financially by strengthening coordination, carrying out a rapid assessment of the injecting drug use situation, organizing study tours, developing and adapting guidelines for the treatment of addiction, organizing advocacy workshops and supporting the secretariat of the Task Force.

The main partnership of WHO in the country is with the Sweden Cooperation for a 2 years project (2008/2009) specifically dedicated to the implementation of Harm Reduction as a key strategy to avoid or mitigate the epidemic in Lao PDR.

As Lao PDR does not yet have an HIV epidemic caused by injecting drug use, this gives the country the opportunity to start a meaningful harm reduction programme and thereafter try to prevent an epidemic.

### Malaysia

#### The epidemic

The first case of HIV was diagnosed in Malaysia in 1986 [54]. By December 2007, the estimated number of PLHA in Malaysia was 69,000 out of a total population of 25,347,000 [55]. The majority of reported cases were in the age group of 20–39 years, the younger and potentially more productive segment of the country's population. During the same period, there were 76,389 reported HIV infections of which 70,300 were in males and 6089 in females (cumulative); 73.7% of HIV infections occurred among PWID [56].

As a consequence of the large proportion of PWID in the population with HIV/AIDS, the epidemic in Malaysia is clearly a concentrated epidemic, since HIV prevalence has been less than 1% among the general population but consistently higher than 5% (between 3% and 20%) in PWID [57]. The first round of the national BSS conducted during 2003–2004 showed a high frequency of sharing injecting equipment among PWID (71.5%) [55]. Males account for more than 90% of the reported cases; however, the epidemic is rapidly advancing among females [55,56]. The proportion of reported HIV infections transmitted through homo/bisexual and heterosexual contacts is also increasing [55]. There is a high level of prevalence in specific populations and certain regions, as more than 10% of commercial sex workers in Kuala Lumpur were found to be positive for HIV in a study conducted in the year 2000.

#### Response to the epidemic in Malaysia

From a highly punitive approach of sending drug users to forced treatment centers as the only strategy by the beginning, the response to the HIV/AIDS epidemic in Malaysia has been stepped up in the past 18 months [58]. Responsibility for drug treatment rests since 2006 with the Ministry of Health, and Malaysia's National Strategic Plan on HIV/AIDS for 2006–2010 includes calls for methadone and buprenorphine, needle exchange and free ART [59]. Malaysia now has six NSPs coordinated by the Malaysian AIDS Council (MAC) [58]. These projects handle more than twice the expected number of clients. At the end of 2007, 3600 PWID had been reached by NSPs [58]. A target has been set of reaching 20,000 PWID by 2010 [58].

MMT has been scaled up continuously since it was introduced in October 2005. The country now has 74 MMT services serving more than 4000 PWID. As of 31 December 2007, a total of 4,135 drug addicts had been registered and enrolled into the Government MMT programme, which is totally free for patients. Furthermore, the expansion of methadone availability in Malaysia is being also amplified by the inclusion of General Practitioners. In April 2008, the MMT programme was extended into the prison system starting with a pilot project in Pengkalan Chepa prison in one of the eastern states. Methadone availability is being enhanced and monitored by the Ministry of Health. The cost has been reduced several fold, but qualified counsellors are scarce [58].

At present, 40% of those who fulfill the criteria for initiating ART are under treatment (personal communication, Dr Christopher KC Lee, during the WHO symposium on Drug use and HIV in Kuala Lumpur, Malaysia, 3 December 2007). This is a great development in comparison with 10% 18 months ago (personal communication, Dr Christopher KC Lee, during the WHO symposium on Drug use and HIV in Kuala Lumpur, Malaysia, 3 December 2007). However, Malaysia still needs to develop the sexual component of its response to the HIV/AIDS epidemic due to the rapid spread among the female population.

#### The role of WHO in Malaysia

WHO has been constantly and consistently providing technical support for capacity building. The first contribution to the Government of Malaysia in this field was in 2004 when an Injecting Drug User Behaviour Survey Study was conducted with technical and financial support from WHO. This study was a baseline for a series of decisions that the Government took to better develop its strategy to confront the epidemic.

In September 2004, a workshop on the development of the National Strategic Plan on HIV/AIDS was conducted, again with WHO support. The Plan was developed in the workshop itself and the first draft was designed by a WHO consultant to support the Government of Malaysia. This strategy was later reviewed in 2005, also with WHO support, when the Government of Malaysia presented their evaluation of the Millennium Development Goals (MDGs) and regretted that the only goal that Malaysia did not achieve at that point in time was MDG 6 – failure to control HIV/AIDS.

Again in 2005, in conjunction with the Asian Parliamentarian Forum, WHO supported a dialogue in the Malaysian Parliament advocating for their support to harm reduction. This activity brought about strong political support for harm reduction.

In 2006, WHO conducted a Bi-Regional Workshop (WPRO and SEARO) in Malaysia and an Informal Consultation to develop guidance for care, support and treatment for PWID. This was crucial for developing the next step of engaging PWID in treatment with ARVs.

Technical support was also provided for the monitoring and evaluation exercises associated with the pilot NSP and MMT programmes. WHO Malaysia hired highly respected international consultants to help conduct an in-depth analysis of the MMT as well as NSP programmes. There was no seminar or event in that field where WHO was not clearly involved.

WHO also supported a Rapid Assessment Study among PWID; and their initial findings were presented during the Symposium on Drug use and HIV in Kula Lumpur, Malaysia, held on 3 December 2007. The study concluded that the majority of subjects initiated drug use before the age of 18 years and shift to injecting very fast. Heroin is the main drug of injection; however, an increasing number of people use ATS as their main drug of choice. Rates of sharing of injection equipment are very high and condom use is less than 20% with all kinds of partners. The data will be released in 2008 by WHO and the University of Sains, Malaysia.

The country's response to the HIV/AIDS epidemic among PWID was analysed in depth at a national AIDS conference and a national symposium on Drug use and HIV promoted by WHO and partners in December 2007. The achievements were acknowledged both within and outside Malaysia. Malaysia's understanding of its epidemic pattern is increasing based on evidence, and the country is stepping up its response by scaling up methadone clinics, NSPs, as well as care, support and treatment for PWID.

At present, besides ongoing technical assistance, WHO is providing support for policy development in the area of Government and public acceptance of harm reduction programmes.

### The Philippines

#### The epidemic

The HIV epidemic in the country has been described as low prevalence [60]. There were 7,490 people estimated to be living with HIV in 2007, out of an adult population of 44,608,300 (15–49 years old), with an HIV prevalence rate of 0.017%. HIV in the Philippines is predominantly sexually transmitted (85%) [60].

However, drivers of the epidemic in PWID have been documented. In 2007, an integrated HIV behavioural and serological surveillance (IHBSS) showed that 52% of PWID had shared needles and syringes the last time they injected; condom use was only 24%; and prevalence of hepatitis C among PWID was 88% in Cebu City and 6% in Zamboanga City [61]. At present, there are about 9,984–20,316 PWID in the country [62].

The National HIV and AIDS Registry, a passive form of surveillance, recorded a total of 3,061 reported cases of HIV from January 1984 to December 2007, of whom 7 were PWID [62].

#### Response to the epidemic in the Philippines

The Department of Health (DOH), as chair of the Philippine National AIDS Council, a multisectoral policy-making body on HIV prevention, is the lead government agency mounting a national response to the HIV epidemic. The country's responses are geared towards universal access to prevention, treatment, care and support, including protecting the rights of PLHA and their families [63].

The following prevention strategies are being implemented: community outreach and education, scaling-up counselling and testing, early diagnosis and management of STIs, 100% condom use programme (15 sites), prevention of mother-to-child transmission (PMTCT, pilot implementation), provision of post-exposure prophylaxis, and a harm reduction programme for PWID (3 sites) [64].

Implementing a harm reduction programme in the Philippines is challenging because of the illegal nature of drug use. Despite a law on the prevention and control of AIDS (Republic Act 8504), a more recent law, the Dangerous Drugs Act (Republic Act 9165) emphasizes the supply and demand reduction approach to control drugs. Recognizing these conflicting mandates, the National AIDS STI Prevention and Control Programme and the Philippine National AIDS Council (PNAC) Secretariat initiated discussions with the Dangerous Drugs Board, the Philippine Drug Enforcement Agency, and other relevant partners, to come up with more appropriate and acceptable approaches that would treat the PWID situation in the country as a public health concern to be addressed rather than as a criminalized, legal issue.

While central government agencies tried to work at the policy level, NGOs, in coordination with the local health departments and local stakeholders, started providing harm reduction services among PWID including community outreach, peer education, referral networking to counselling, provision of clean needles and syringes, psychosocial support, and referral and treatment for STIs and other health-related concerns. Outreach work was initiated in 1995 by the Program for Appropriate Technology in Health (PATH) in partnership with the University of Southern Philippines Foundation, through the AIDS Surveillance and Education Project of USAID. Other NGOs such as Remedios AIDS Foundation, KABALIKAT, USAID-Local Enhancement and Development (LEAD) for Health Project were also involved in outreach education work among PWID. Currently, the Philippine NGO Council for Population, Health and Welfare, Inc. and the Tropical Disease Foundation continue to implement PWID harm reduction programmes through the Global Fund AIDS projects.

In 2007, the Asian Development Bank's Regional Technical Assistance Project complemented the country's initiatives by supporting the conduct of a situation and response analysis of PWID-related work in the country and of IHBSS among PWID in the cities of Zamboanga and General Santos [65].

Currently, 800 PWID are reached by prevention services through three major outreach service delivery points, which includes provision of clean needles/syringes [64,66,67].

In the area of care and support, there are 11 treatment hubs with trained HIV AIDS Core Teams that provide clinical and psychosocial care and support to PLHA. ART is provided free since 2005. The baseline CD4 count is also done free at the STI AIDS Cooperative Central Laboratory (funding support from Global Fund). As of December 2007, there are 390 PLHA enrolled for free ART and are receiving extended care and support services [64].

#### Role of WHO

WHO remains the major provider of assistance to the DOH in advocating for sustained efforts to support the harm reduction programme among PWID and to dispel the perception that this is not effective and not needed in the Philippines. WHO is the lead agency on matters pertaining to health, and in preventing the transmission of HIV through injecting drug use (since there is no UNODC office in the Philippines). In conjunction with other UN agencies, WHO supports the evidence-based development of harm reduction strategies and ensures that only scientifically effective harm reduction strategies are introduced, adapted and continuously implemented in the Philippines in order to address the public health concerns of people who inject drugs.

WHO supported a study on behaviour patterns among PWID admitted to rehabilitation centres; the results were initially disseminated in January 2008. WHO also conducted the first orientation on PWID harm reduction programme (2007) among DOH and UN staff, and among high-level officials of the DOH, Department of Interior and Local Government, and the PNAC Secretariat. WHO is a key member of the Harm Reduction Working Group which will draft appropriate national guidelines on the harm reduction programme in the Philippines.

### Viet Nam

#### The epidemic

After the first HIV case was diagnosed in 1990, transmission and reporting of cases accelerated such that it was estimated that there were 293,000 PLHA in Viet Nam at the end of 2007 [68]. Cumulative reporting up to end of December 2007 documented 156,210 HIV infections; 62,145 cases of AIDS and 34,476 deaths due to AIDS [69]. The majority of the reported cases (83.16%) were aged between 20 and 39 years; 57.28% of the total were between 13 and 29 years; 82.77% of cases were male and prevalence among women was slowly increasing [68]. Estimated adult HIV prevalence is 0.5% [70].

Since 1990, the majority of reported HIV infections and AIDS cases have been in PWID (50–60%), though heterosexual transmission, particularly through commercial sex, appears to be increasing [69]. With a low prevalence of HIV in the general population and high prevalence in PWID (28.6% nationally), the HIV epidemic remains "concentrated" [68]. In the Viet Nam Integrated Behavioural and Biological Surveillance Survey of 2005–2006, around one third of PWID reported sharing of syringes in the previous six months and more than 50% reported unprotected sex with sex workers [71].

#### Response to the epidemic in Viet Nam

The Ministry of Health (MoH) is the lead agency for harm reduction activities. HIV prevention measures were initiated in 1993 and predominantly involved mass education and small-scale needle/syringe distribution. In 2003, the first of the large donor-funded prevention projects started with financial support from the UK Department for International Development (DFID) and Norwegian Directorate for Development Cooperation (Norad) in 19 provinces and two cities (initially under MoH-WHO co-managment, later by MoH with technical assistance from WHO). In 2005, the response was expanded by the addition of the World Bank-funded MoH Viet Nam HIV/AIDS Prevention Project in 18 provinces and two cities (six provinces and the two cities overlap) [72].

An impressive government HIV/AIDS response over the past five years has seen an expanding public health response and the National Assembly pass the Law on HIV/AIDS Prevention and Control in 2006 and associated Decree 108/2007 ND-CP in 2007 [73,74]. Protracted and careful advocacy on the part of the Communist Party Commissions, local NGOs, international community members and dedicated National Assembly members facilitated passage of the 2006 Law.

Under the Law, the MoH is specifically mandated to lead harm reduction activities for HIV prevention among risk groups and to work with the Ministry of Public Security (MoPS) and Ministry of Labour, Invalids and Social Affairs (MoLISA) to ensure the implementation of needle and syringe, condom distribution and OST programmes.

The MoH Viet Nam Administration of HIV/AIDS Control (VAAC)-led projects for prevention have expanded since the HIV/AIDS Law, in particular, coverage with NSPs increased from 21 provinces in 2005 to 42 provinces in 2007. During 2007, expansion of all projects led to the distribution by government health services of more than 11 million needles and syringes (sufficient to provide around one quarter of the registered PWID with one per day), and more than 100 million condoms – predominantly through the activities of more than one thousand peer outreach workers [75,76].

In 2007, VAAC, in collaboration with other MoH departments and international partners, developed national guidelines and a proposal for pilot MMT programmes for 1500 patients. Treatment commenced at three sites each in Hai Phong and Ho Chi Minh City during the first half of 2008 with support from the US Government President's Emergency Plan for AIDS Relief (PEPFAR), WHO, DFID and World Bank [77].

By the end of September 2007, a total of 14,180 adults were receiving ART in 64 provinces – a 5.7-fold increase from the end of 2005 (or 28.4% of those needing ART) [69]. Though the proportion of ART patients who are former or active PWID is unclear, scaling up of ART created opportunities for direct collaboration between health staff and PLHA support groups whose risk behaviour had previously marginalized them. This was important for mobilization of appropriate peer workers for outreach in harm reduction.

The national network has more than 80 Treatment and Education Centres, which are institutions for drug users (and sex workers) who are required to go through drug detoxification, education and occupational training for one to two years (except in Ho Chi Minh City which has a five-year requirement). Implementation of small-scale provision of ART commenced this network in 2007 with PEPFAR support. Substantial expansion is planned under the Global Fund Programme in 2008 and beyond. Many of these closed settings, housing over 60,000 drug users, have a high prevalence of HIV (30–60%) and burden of AIDS-related illnesses, but until now have been without adequate resources to provide HIV prevention, treatment or care [69].

#### The role of WHO in Viet Nam

In response to requests from the MoH, WHO has been a principal provider of technical assistance for harm reduction activities, which are largely conducted by the health sector. WHO efforts to increase the coverage and quality of harm reduction interventions for the MoH-DFID/Norad-funded HIV prevention project in 21 provinces has been through the development of guidance documents, tools, identification of good practices for the needle-syringe and condom programmes for entertainment establishments, street-based sex workers and other groups at risk through non-pharmacy outlets, and capacity building, including participatory training for managers and peer outreach workers.

Building on these experiences, WHO has been taking a lead role in supporting VAAC, with support from DFID and SIDA, to develop the national technical guidelines on the needle-syringe and condom programmes, in collaboration with PEPFAR, UNAIDS, UNODC and others. Furthermore, development of strategic and operational collaboration between MOH, MOLISA, MOPS and their local entities are being promoted and supported by Joint UN Team on HIV in which WHO plays an active role.

Through 2007, WHO and international partners supported the complex collaborative development process for the MMT guidelines, implementation proposal, training curriculum and methadone procurement.

WHO provided key support to OST/NSP study tours for the MoH in 2007 to sites in Hong Kong, Malaysia, Indonesia and Australia, including participation to provide translation into a familiar language and organizational context. WHO continues to collaborate with UNODC and UNAIDS to ensure that partnerships between the MoH and MoPS/MoLISA remain supportive of harm reduction.

WHO has continued to support expansion of HIV treatment and care including ART for active PWID and development of the National HIV Monitoring and Evaluation Framework including refinement of indicators related to PWID and Sex Work.

Challenges remain for WHO, working with advocacy partners, to expand the supportive political environment for harm reduction in Viet Nam and in the development of effective HIV prevention and care linkages between closed settings ('Treatment and Education' centres/prisons) and communities.

### WHO Regional Office for the Western Pacific (WPRO)

In 2003, the WHO Region for the Western Pacific and Asia developed a workplan in support of harm reduction that embraced HIV/AIDS prevention, care, support and treatment for PWID. It was based on assessment missions to countries with varied situations with regard to HIV and the use of drugs including China, Cambodia, Lao PDR, Malaysia and Viet Nam. Consultations were also held also with key partners in this area, including UNAIDS, UNODC, CDC-Global AIDS Program, Family Health International (FHI) and Burnet Center for Harm Reduction. The framework used reflects WHO's overall management plan and expected results: to develop tools and guidelines; to strengthen country capacity; and to provide regional support for country programme development.

One of the expected results was the development of tools and guidelines:

(1) A publication entitled *Drug dependence detoxification and treatment guidelines for low-resource, high HIV-risk settings*. These protocols and associated training packages have been developed as a joint initiative with the Australian National Drug and Alcohol Research Centre, one of the WHO Collaborating Centres. The package is being field-tested in Cambodia in the second semester of 2008. A final version will be published by the end of 2008.

(2) A manual on *HIV/AIDS care and treatment for people who inject drugs *was published in March 2008.

(3) *HIV testing and counselling in settings attended by people who inject drugs *is scheduled for publication in 2008.

These tools and guidelines will be extremely helpful for Members States and civil society to further develop harm reduction strategies in the Region.

(4) A revised framework to address TB-HIV Co-Infection in the Western Pacific Region, was launched in July of 2008 and dedicated one chapter for TB-HIV in Closed Settings and among people who inject drugs.

A second main task from the workplan is to give support to Member States. It is important to state that HIV Programmes in WHO country offices in the region count with a focal person for Harm Reduction in the six mentioned countries. Their contribution is being crucial. Consistency and technical back-up for the work of WHO country offices in the Region is provided by WPRO. The HIV/STI Focus Unit of WPRO had an important role to play in the achievements made by the six countries discussed in this paper.

The third main task is to provide regional support. A standing participation in the UN Regional Task Force has been an important contribution from WHO to a collective effort. WPRO is also working hard to develop regional alliances with crucial partners (UNODC, UNAIDS, UNICEF, DFID, AusAID, SIDA, WHO/SEARO, International Network of People Using Drugs [INPUD], and others) for better development of harm reduction strategies to tackle the epidemics in the Region. Together with UNODC, WPRO will organize a meeting during the 20^th ^International Harm Reduction Conference in Bangkok, in 2009, of all the relevant community-based organizations in the field of drug use. WPRO is also collaborating with the Australian National Council on Drugs and the Burnet Institute to better develop a network on drug research in the Pacific Area, almost unexplored in terms of the health consequences of the misuse of illegal drugs.

At present, the main objectives of the WPRO HIV/AIDS and STI Unit in the field of harm reduction are to harmonize (as much as possible) the response to the HIV/AIDS epidemics among PWID in different countries of the Region; help countries to develop better policies, legislations and practices to facilitate their response; increase the availability of advocacy tools; reinforce the main package of interventions comprising NSP, Drug Dependence Treatment (mostly but not only OST) and care, support and treatment (including ARV). Co-infections of TB and Hepatitis C are also being addressed. WHO is in a privileged position among the UN agencies to be the interlocutor with the health sector, supported by science, evidence-based results and political will. WHO also has the technical expertise to take the lead in helping to promote the changes needed to adequately face the epidemic among and from people who inject drugs.

## Conclusion

Injecting drug use is driving HIV epidemics in many countries and accounts for almost a third of new infections outside sub-Saharan Africa [3]. Across the estimated 13 million PWID globally, drug use patterns, behaviours and contexts vary widely [78]. The common thread that runs through all epidemic situations in the Asia-Pacific region is that the major HIV risk behaviour groups affected (PWID, MSM, sex workers and their clients) are socially marginalized and engage in socially unacceptable and often illegal behaviours [22]. Many countries in Asia face difficult public policy and legislative problems with regard to sex work, homosexuality and drug use. In addition, widespread poverty, and a general lack of access to effective health and welfare services by the poor and disadvantaged in both rural and urban areas, means that the challenges of developing targeted intervention programmes, and ensuring coverage of vulnerable groups, are particularly acute. Attention to these challenges is urgently required [78]. Supportive policies, including policies that ensure equitable access to HIV services for drug users; a conducive legal and social environment; laws that do not compromise access to HIV services for drug users through criminalization and marginalization; and campaigns to reduce stigma and discrimination, are needed to combat the epidemics [78]. Until there is full public and policy-maker acceptance of the need to develop and expand effective risk behaviour change, and reduction or elimination programmes, HIV will continue to spread in the Asia-Pacific region [21].

In conjunction with many other partners, WHO pioneers the promotion and development of evidence-based strategies for harm reduction in the Region. Because of the efforts made, harm reduction is no longer a marginal strategy in the Region. While the progress made has been impressive, it is important to consolidate the advances and scale up interventions to better confront the epidemics driven by injecting drug use.

## Competing interests

The authors declare that they have no competing interests.

## Authors' contributions

DJ and MF were responsible for the Vietnam piece of the paper. DR developed the Lao PDR piece of the manuscript. GS was the main contributor for the Cambodian piece of the paper. HT and NS contributed to the piece of the paper on Malaysia. HY and KP were the main responsible for the piece of the paper about China. MS was in charge of the piece of Philippines in the manuscript. FM as the main author overviewed the paper and was responsible for the overall manuscript. All authors read and approved the final manuscript.
